# Survival of a newborn from a pregnant woman with rabies infection

**DOI:** 10.1186/s40409-016-0068-5

**Published:** 2016-04-02

**Authors:** Zhen-Yu Qu, Guo-Wei Li, Qiao-Ge Chen, Peng Jiang, Chang Liu, Alfred Lam

**Affiliations:** Department of Pathogenic Biology and Immunology, Luohe Medical College, Luohe, Henan 462002 PR China; Department of Prevention and Control of Infectious diseases, Zhengzhou Center for Disease Control and Prevention, Zhengzhou, Henan 472000 China; Department of Anatomy, Henan Medical College, Zhengzhou, Henan 472000 China; Department of Pathogenic Biology, Zhengzhou University, Zhengzhou, Henan 472000 China; School of Medicine and Griffith Health Institute, Griffith University, Gold Coast, Australia

**Keywords:** Rabies, Pregnant, Newborn baby

## Abstract

**Background:**

Rabies is very common in People’s Republic of China. Each year thousands of people die because of this disease, but rabies diagnosed in pregnancy is very rare.

**Case Presentation:**

In this study, we report the case of a pregnant woman who was infected with the rabies virus after a dog bite. The symptoms of rabies appeared in labor and she died after pregnancy. Her baby and husband did not develop the disease.

**Conclusion:**

The phenomenon that the newborn infant was healthy may be related to the protective role of placenta in resisting the invasion of the rabies virus or the absence of systemic viremia. The prompt administration of vaccines and anti-rabies immunoglobulin to the infant may have also contributed to his survival.

## Background

Whereas rabies has a mortality rate of nearly 100 %, China remains a high-risk region for its transmission. During the period from 2006 to 2011, the numbers of deaths due to rabies nationwide in China were 3293, 3303, 2466, 2213, 2048 and 1917, respectively [1]. The diagnosis of rabies during pregnancy is extremely rare. To the best of our knowledge, five cases have been recorded in the literature in which the newborn infants survived [2–6]. On the other hand, in a case reported in Turkey, a full-term baby died of the disease [7] (Table 1). In this report, we report the case of a pregnant woman who was infected with rabies virus after a dog bite. The symptoms of rabies appeared in labor and she died after birth. Up to the present moment, the baby and husband were free of the disease after treatment.

**Table 1:** Reported rabies cases in pregnant women and neonatal outcome

Study	Year	Reported area	Neonatal outcome
Mondal et al. [2]	2014	Nigeria	Survival
Swende and Achinge [3]	2009	Cameroon	Survival
Iehlé et al. [4]	2008	Thailand	Survival
Lumbiganon and Wasi [5]	1990	Turkey	Survival
Sipahioğlu and Alpaut [7]	1985	German	Death
Müller-Holve et al. [6]	1977	India	Survival

## Case presentation

In May 2013, a 25-year-old, 4-month pregnant woman was bitten by a dog on the right instep when wearing slippers and walking on the Xiaoying village road. The place is located 40 km northwest of PingDingShan city, Henan Province, PR China. The patient went to the village health clinic to see a general practitioner because the bite site was bleeding. The nurse disinfected the wound with iodine and alcohol and no measure concerning rabies immunization was taken. Three days later, the wound had healed.

Months later, on November 6, the woman was in the delivery room in Baofeng Hospital of Traditional Chinese Medicine in Pingdingshan city. On that night, she felt pain on the right leg as well as agitation and insomnia. On the following day (November 7) at 8:30 a.m., she gave birth to a baby boy in the emergency room by caesarean section. The birth weight of the infant was 2.5 kg and he was healthy according to all relevant indexes. During the procedure, although the woman was shivering, she was conscious and her blood pressure was within the normal range. At 5:00 p.m. on November 7, she started to develop symptoms such as thirst, headache, fear, chest distress, hidrosis and difficulty in swallowing water. The doctor on duty prescribed sedatives for her in order to ameliorate the symptoms. On November 8, manifestations including hydrophobia, vomiting, madness, dysphagia and fear appeared. She was clinically diagnosed with rabies. On November 9, the patient was transferred to the Infectious Diseases Hospital of Henan Province.

On physical examination, the patient had low fever (temperature: 37.5 °C). She was conscious but with intermittent irritability. There was no palpable lymph node. Examination of the cardiovascular and respiratory system was unremarkable. The abdomen was soft and with an operative incision about 10 cm long seen in the hypogastrium. The dressing was dry with no bleeding. The patient was isolated quickly in the hospital. The nurse used diazepam to sedate the patient who was having obvious symptoms of irritability.

One day after the cesarean section, she was given a combination of antibiotics, piperacillin and tazobactam as well as anti-viral drug (ribavirin) to treat the infection. In addition, mannitol was given to reduce intracranial pressure. Moreover, two Chinese medicines, Xuebijing injection (intravenously, 50 mL plus 0.9 % 100 mL saline, two times a day) and Xingnaojing injection (intravenously, 10 mL plus 0.9 % 250 mL saline, two times a day) were given. Xuebijing injection is a complex traditional Chinese prescription consisting of flos *Carthamus*, radix *Paeonia* rubra, Chuanxiong rhizoma, radix *Salvia miltiorrhiza* and radix *Angelica sinensis* and was noted to help to improve blood microcirculation, to avoid toxin spreading in suppurative infections and ti inhibit release of tumor necrosis factor-α. Xingnaojing injection, which is composed of four medicinal herbs – including artificial musk, synthetic borneol, *Curcuma aromatica* Salisb. and *Gardenia jasminoides* J. Ellis – is a well-known traditional Chinese patent medicine (TCPM) and is believed to reduce brain injury and enhance functional recovery after stroke [8, 9].

Despite treatment, the symptoms got progressively worse. The patient frequently spitted saliva and could not lie down because of chest tightness. She was immobilized and receiving intravenous injection of diazepam. Her symptoms did not improve. The family requested discharge against medical advice. At 12:00 p.m. on November 9, the patient died at home in respiratory failure. Her husband and baby boy were diagnosed with class III rabies exposure according to WHO classification (single or multiple transdermal bites or scratches, contamination of mucous membrane with saliva from licks; exposure to bat bites or scratches). At 4:00 p.m. on November 10, the husband and baby received rabies vaccine (Vero cell) by intramuscular injection in a traditional Chinese medical hospital of Baofeng county. Four further doses were given at days 3, 7, 14, and 28. They were free of the disease up to September 30, 2015.

## Conclusions

According to previous studies, hundreds of pregnant women are exposed to injuries provoked by dog in China every year [10, 11]. Nearly all patients in urban areas in China had received post-exposure prophylaxis (PEP) which was effective and safe. However, some people exposed to rabies, especially in rural areas, are not vaccinated because they ignore the danger of rabies and cannot afford the expensive vaccine. In addition, there are still many dogs in these areas without management. Because of the above reasons, rabies has been a serious threat to the local population.

This study reports the case of a pregnant woman who did not receive PEP and the symptoms of rabies appeared in labor. During this period, the virus may have spread in vivo. The newborn baby was healthy may due to the absence of systemic viremia or because of the protective role of the placenta.

Although laboratory diagnosis comprises an important role in the management of rabies infection, most cases of the disease occurs in rural areas of China, where no health facilities have technical conditions for the detection of the virus. The government should set up monitoring centers for laboratory identification of rabies virus in areas of high incidence in order to prevent its transmission. Latin American countries were almost able to eradicate rabies by strengthening measures against its virus through mass vaccination of dogs, for example [12, 13]. These successful experiences suggest that it is possible to fight the disease by means of virus control and prevention.

### Consent

Written informed consent was obtained from the victim’s husband for publication of this article.
